# Lightweight GAN for Restoring Blurred Images to Enhance Citrus Detection

**DOI:** 10.3390/plants14193085

**Published:** 2025-10-06

**Authors:** Yuyu Huang, Hui Li, Yuheng Yang, Chengsong Li, Lihong Wang, Pei Wang

**Affiliations:** 1 Key Laboratory of Intelligent Agricultural Equipment in Hilly and Mountain Areas, College of Engineering and Technology, Southwest University, Chongqing 400715, China; yu1174130407@email.swu.edu.cn (Y.H.); leehui@swu.edu.cn (H.L.); l20190304@swu.edu.cn (C.L.); w20190305@swu.edu.cn (L.W.); 2 Key Laboratory of Low-Carbon Green Agriculture in Southwestern China, Interdisciplinary Research Center for Agriculture Green Development in Yangtze River Basin, Southwest University, Ministry of Agriculture and Rural Affairs, Chongqing 400715, China; yyh023@swu.edu.cn; 3 National Citrus Engineering Research Center, Chinese Academy of Agricultural Sciences & Southwest University, Chongqing 400712, China; 4 Key Laboratory of Agricultural Biosafety and Green Production of Upper Yangtze River, College of Plant Protection, Southwest University, Ministry of Education, Chongqing 400715, China

**Keywords:** image deblurring, AGG-DeblurGAN, lightweight, image quality assessment, citrus detection

## Abstract

Image blur is a major factor that degrades object detection in agricultural applications, particularly in orchards where crop occlusion, leaf movement, and camera shake frequently reduce image quality. This study proposed a lightweight generative adversarial network, AGG-DeblurGAN, to address non-uniform motion blur in citrus tree images. The model integrates the GhostNet backbone, attention-enhanced Ghost modules, and a Gated Half Instance Normalization Module. A blur detection mechanism enabled dynamic routing, reducing computation on sharp images. Experiments on a citrus dataset showed that AGG-DeblurGAN maintained restoration quality while improving efficiency. For object detection, restored citrus images achieved an 86.4% improvement in mAP@0.5:0.95, a 76.9% gain in recall, and a 40.1% increase in F1 score compared to blurred images, while the false negative rate dropped by 63.9%. These results indicate that AGG-DeblurGAN can serve as a reference for improving image preprocessing and detection performance in agricultural vision systems.

## 1. Introduction

In recent years, computer vision and deep learning-based object detection technologies have been widely applied in agriculture, showing great potential for tasks such as pest and disease monitoring, fruit recognition and harvesting, growth assessment, and autonomous navigation [1,2,3,4]. Citrus, as a representative economic fruit crop, is cultivated on a large scale and requires complex field management, which places higher demands on automated perception systems. During operations such as spraying and harvesting, dense foliage, varying lighting conditions, frequent occlusions and movements, and mechanical vibrations often cause non-uniform blur in captured images. Such blur reduces the accuracy of subsequent detection and recognition tasks [5,6]. Therefore, addressing the complex blur in citrus images is crucial for improving the robustness and practical value of intelligent agricultural perception systems.

In dynamic agricultural scenes such as citrus orchards, non-uniform motion blur caused by relative movements among the camera, crops, and background is one of the most common types of image degradation. Under conditions of dense foliage and irregular fruit distribution, blur often exhibits spatial nonlinearity and local inconsistency, making it difficult for traditional image enhancement methods to handle effectively. Image deblurring algorithms can effectively restore blurred images [7,8]. Depending on whether the blur kernel is known, deblurring approaches can be divided into blind and non-blind categories [9]. Traditional methods such as the Richardson–Lucy algorithm based on maximum likelihood estimation, Wiener filtering, and Markov random fields rely on handcrafted priors and struggle to handle the diverse and complex blur patterns in citrus images, while also suffering from low processing efficiency [6,10]. In recent years, deep learning has enabled effective deblurring by modeling complex blur kernels or learning direct mappings between blurred and sharp images [6,11].

Previous research has shown that blind deblurring methods tend to perform better, while non-blind approaches depend on precise blur kernels, and inaccurate parameters can compromise restoration performance [12]. In complex agricultural environments, estimating spatially nonlinear and non-uniform blur parameters remains highly challenging. Blind deblurring methods that do not depend on kernel accuracy are particularly important, such as deep learning-based image-to-image models. Generative adversarial networks (GANs) have addressed the limitations of conventional feed-forward networks in loss modeling by employing adversarial learning, thereby enabling the generation of sharper and more structurally consistent images [13]. DeblurGAN is an end-to-end GAN-based model capable of restoring clear images from both synthetic and real-world blurred inputs [14], while its enhanced version, DeblurGAN-v2, further improves deblurring performance and inference efficiency [15]. More recently, the Restormer model, based on Efficient Transformers, demonstrated state-of-the-art performance in image restoration tasks [16]. End-to-end deblurring methods have also shown strong potential in agricultural applications. Shah and Kumar [17] applied SRN-Deblur, Deep-Deblur, and DeblurGAN-v2 to grape images affected by motion blur, where the mAP increased from 0.196 to 0.233, 0.263, and 0.309, respectively, demonstrating improved detection accuracy and differences among models in handling blur. Xing et al. [5] proposed a multi-scale feature extraction network (MFENet) to address blur caused by branch movement during wolfberry harvesting, achieving a 36.72% improvement in average precision for branch recognition. In addition, end-to-end nonlinear deblurring methods based on GANs have been used to restore images of weeds and specific crops blurred by camera shake or plant motion, leading to significant improvements in recognition accuracy [18,19]. Despite these advances, challenges remain in motion deblurring, particularly in model interpretability, blur parameter estimation accuracy, robustness, and computational efficiency.

This study focused on citrus trees and proposed an improved lightweight GAN to address motion-induced image blur during spraying or harvesting operations. The aim was to enhance object detection accuracy and provide technical support for precision agriculture based on vision and imaging technologies. The main contributions are summarized as follows:

(1) To the best of our knowledge, few studies have focused on motion deblurring in orchard environments. In this work, an improved lightweight GAN-based model was proposed for citrus trees to restore motion-blurred images while reducing model complexity and improving efficiency.

(2) A set of image quality assessment metrics, including DISTS, LPIPS, VSI, FSIM, MS-SSIM, GMSD and NLPD, was employed to objectively evaluate the quality of deblurred citrus images and quantitatively measured deblurring performance.

## 2. Materials and Methods

### 2.1. Construction of the Citrus Image Dataset

#### 2.1.1. Sharp Image Acquisition

Sharp images were collected at the citrus plantation of the Research Base of Southwest University, Chongqing, China, using the front-facing camera of an iPhone 13. Videos of citrus trees were recorded at a resolution of 1920 × 1080 and a frame rate of 60 fps in MOV format. Frames were extracted at intervals of 30 frames, and all extracted images preserved the original resolution, resulting in 1000 sharp images. To expand the set of sharp samples, data augmentation techniques were applied, including horizontal flipping, random rotation, and brightness adjustment. These transformations produced 3000 additional sharp images with variations in spatial orientation and illumination. In total, 4000 sharp images were obtained, all maintained at the resolution of 1920 × 1080 for subsequent blur synthesis, training, and inference.

#### 2.1.2. Dataset Synthesis and Pairing

In this study, a two-dimensional trajectory generation method based on simulated random camera motion was proposed to synthesize realistic motion-blurred image data. Motion paths of varying complexity were generated on a two-dimensional plane to mimic camera shake or nonlinear movements, with the blur intensity parameter controlling path complexity. The motion range was constrained by the image scale, and paths were incrementally generated by randomly sampling step lengths and angles. Step length determined the displacement of each movement, while angle defined the turning direction. As blur intensity increased from 0 to 1, trajectories shifted from near-linear to highly random. Step lengths were sampled from a Beta distribution, with higher intensity producing longer average steps. Angles were bounded by the blur intensity and further perturbed by a sign-flip rule to introduce jitter. These increments were accumulated according to Equation (1) to form a complete trajectory in the complex plane. The trajectory was smoothed with a Gaussian filter, rasterized to obtain the point spread function, and convolved with each RGB channel of the sharp image to generate the blurred result.

(1) $$z_{j}=\sum_{k=1}^{j}s_{k}e^{i\theta_{k}}\;,\;j=1,2,\ldots ,N$$

where z_j_ is the position at step j in the complex plane, s_k_ is the step length at step k, θ_k_ is the turning angle at step k. N is the total number of steps.

**Figure 1:**
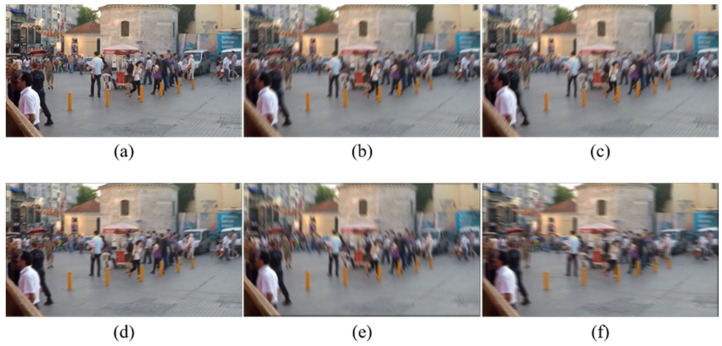
Comparison of blurred images. (**a**) Sharp image. (**b**) Blurred image from the GoPro dataset. (**c**) Gamma blurred image from the GoPro dataset. (**d**–**f**) Blurred images generated by proposed method with blur intensities of 0.25, 0.5, and 0.75, respectively.

To assess the effectiveness of the synthesis strategy, it was applied to sharp images from the GoPro dataset to generate blurred images. The synthesized blurred images were then compared with the paired blurred images provided in the GoPro dataset. The GoPro dataset generates blurred images by averaging consecutive frames captured during. As shown in Figure 1, the synthesized images with blur intensities of 0.25, 0.5, and 0.75 are comparable to both the GoPro blurred images and gamma-corrected blurred images. These results qualitatively indicate that the proposed method can reproduce real-world motion blur with adjustable intensity.

**Figure 2:**
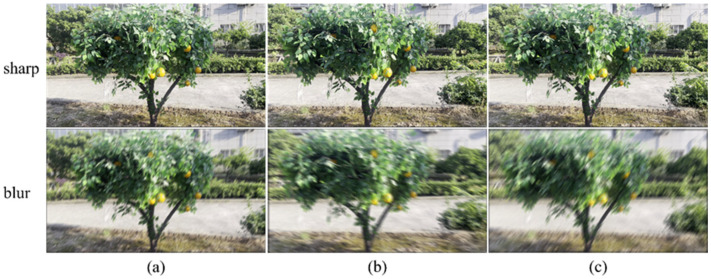
Sharp–blur image pairs. Blur intensity: (**a**) 0.25; (**b**) 0.5; (**c**) 0.75.

In the Citrus Image Dataset, blurred images were generated for each sharp image. The dataset was organized in a paired format of sharp and blurred images and partitioned into training, validation, and test sets in an 8:1:1 ratio. Representative examples of the sharp–blur pairs are presented in Figure 2.

### 2.2. Deblurring Network Model

**Figure 3:**
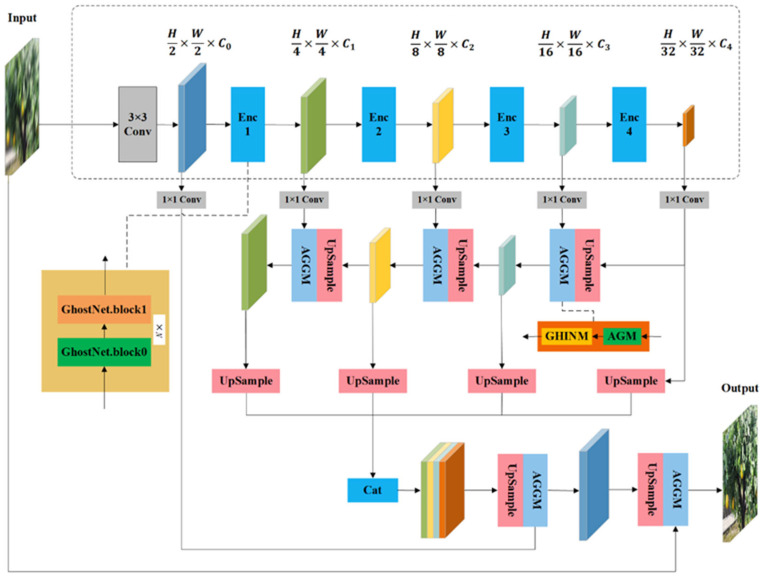
Architecture of the AGG-DeblurGAN generator network.

In this study, a lightweight GAN model, termed AGG-DeblurGAN, was proposed for image deblurring. The model was built upon the DeblurGAN-v2 framework and consisted of a generator and a discriminator. The generator was designed based on a Feature Pyramid Network structure, where GhostNet served as the backbone, and it was enhanced by the integration of an Attention-Ghost Module and a Gated Half-Instance Normalization (GHIN) Module. The overall architecture of the generator is shown in Figure 3.

The network backbone consisted of a 3 × 3 convolutional module followed by four feature encoding modules, where each encoding module contained two GhostNet blocks. The input image was first processed by the 3 × 3 convolution to extract low-level features and then passed through the encoding modules to generate hierarchical feature maps. These maps corresponded to progressively reduced spatial dimensions with channel widths of 16, 24, 40, 80, and 160. To align features from different stages, one-by-one convolutions were applied, yielding lateral features with 64 channels at the shallowest stage and 128 channels at the deeper stages. A top-down pathway was employed to achieve multi-level fusion. The deepest feature map was upsampled by a factor of two using nearest-neighbor interpolation and merged with the adjacent lateral feature map through element-wise addition. The fused map was refined by an Attention-Ghost and GHIN Module. This process was repeated across three successive levels, progressively propagating semantic information from deep layers to shallow ones. The resulting fused features preserved both fine-grained details and high-level context. Each fused feature map was further processed by a two-layer Ghost-based head that reduced its width to 64 channels. These refined maps were then upsampled to the same spatial size and concatenated along the channel dimension, forming a 256-channel tensor that aggregated contextual information across all scales. To strengthen shallow-level compensation, this tensor was combined with the earliest feature map of 64 channels through two successive Attention-Ghost and GHIN Modules, each followed by nearest-neighbor upsampling. This operation ensured consistent integration between low-level structures and high-level semantics. Finally, a Ghost-based convolution reduced the aggregated features to three channels. The output was added to the input image through a residual connection.

#### 2.2.1. AGG Module

In this study, an Attention-Ghost and Gated Half-Instance Normalization (AGG) module was integrated into the generator to improve feature modeling for image deblurring. Within the Ghost module [20], intrinsic features were extracted by standard convolution, and complementary features were obtained by depth-wise convolution. These two outputs were concatenated and processed by a Squeeze-and-Excitation (SE) block [21] before channel cropping. This step adaptively recalibrated channels during fusion.

**Figure 4:**
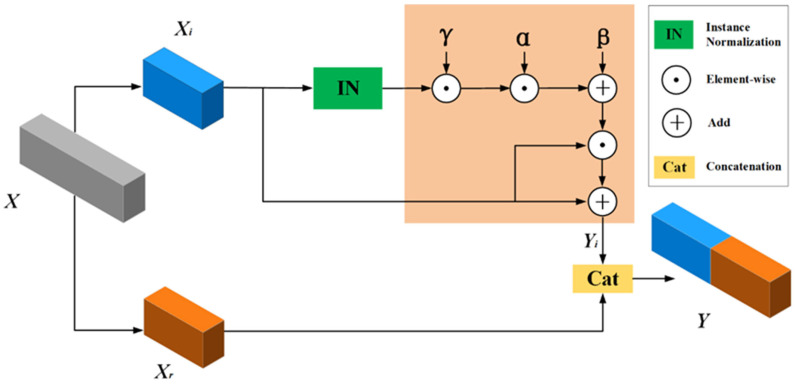
Gated Half-Instance Normalization (GHIN) module.

The GHIN module was inserted at the fusion stages of the top-down pathway. The structure of GHIN module is shown in Figure 4. The GHIN process is shown in Equations (2)–(6). Given an input feature map X∈R^(N×H×W×C), the channels were divided into two parts: the first half X_i_ was normalized, and the second half X_r_ was preserved. The normalized part X_i was standardized and then modulated by a gating mechanism [22]. It used three learnable parameters: scaling gate α, scaling factor γ, and bias β, Each had a shape of 1 × C_i_ × 1 × 1, where C_i_ is the number of normalized channels. These parameters were initialized as α = 1, γ = 0, and β = 0. All parameters were trainable without any activation constraints. This flexibility enabled the network to balance normalization and residual information adaptively, which improved robustness to different levels of blur. The gating mechanism generates a modulation factor, as shown in Equation (4). This factor first multiplies the input X_i_, adaptively modulating the normalized channels. The residual path is then added to the normalized channels to preserve the original information and ensure stability, as shown in Equation (5). Finally, the modulated features Y_i_ and the preserved features X^r^ were concatenated to produce the output feature map Y, as shown in Equation (6).

(2) $$X=[X_{i},X_{r}]$$

(3) $$X_{i}^{norm}=\frac{X_{i}-\mu }{\sqrt{\sigma^{2}+\epsilon }}$$

(4) $$S=\gamma \cdot \alpha \cdot X_{i}^{norm}+\beta$$

(5) $$Y_{i}=(1+S)\cdot X_{i}$$

(6) $$Y=Cat(Y_{i},\;X_{r})$$

where X is the input feature map, X_i_ is the first half of the channels used for normalization, X_r_ is the remained channel, μ and σ^2^ is the per-channel mean and variance, X_i^norm is the standardized feature, ϵ is a small constant, S is the modulation factor obtained through the gating mechanism, α, γ, and β are the parameters of the gating mechanism, Y_i_ is the gated and residual-enhanced output feature of the normalized channels, and Y is the output feature map.

#### 2.2.2. Double-Scale Discriminator

The double-scale discriminator was adopted as the discrimination module of the deblurring GAN. DeblurGAN-v2 proposed a double-scale discriminator that enhanced the ability to distinguish non-uniform blur by integrating information from both global and local discriminators [15]. The global discriminator received a downsampled full image to capture the overall distribution of motion blur and contextual information. The local discriminator focused on high-resolution image patches to analyze fine-grained texture details. By jointly leveraging global structural perception and local texture discrimination, the double-scale discriminator improved the ability to distinguish spatially variant blur, effectively suppressing artifacts such as ringing effects and enhancing the restoration of high-frequency details. This double-scale adversarial mechanism provided multi-granularity feedback to the generator, offering stronger supervision signals that improved both reconstruction quality and training stability.

### 2.3. Loss Function

The overall loss function L_G_ consisted of a content loss L_content_ and an adversarial loss L_adv_. The content loss L_content_ was designed to constrain the generated image in terms of structure, texture, and pixel-level accuracy. It was composed of a perceptual loss L_p_ and L_1_ loss, combined in a weighted form. The formulation of the content loss is defined in Equation (7).

(7) $$L_{content}=0.7L_{p}+0.3L_{1}$$

where $L_{p}$ is the perceptual loss with a weight of 0.7, calculated as the Euclidean distance between the generated image and the corresponding sharp image in the feature space of VGG-19, to capture high-level semantic differences. $L_{1}$ is the pixel-wise absolute error with a weight of 0.3, used to suppress artifacts and color shifts, and to enhance the restoration of fine image details.

To further enhance the naturalness and realism of the generated images, the adversarial loss $L_{adv}$ adopted the relativistic least squares. It was incorporated into the total loss with a relatively low weight to balance adversarial constraints and content supervision. The overall loss function is defined in Equation (8).

(8) $$L_{G}=L_{content}+0.005L_{adv}$$

where $L_{G}$ is the total loss, $L_{content}$ is the content loss, and $L_{adv}$ is the adversarial loss with a weight of 0.005.

### 2.4. Training Parameters

The proposed GAN was implemented based on the Windows 11 system and the PyTorch framework. The training hardware configuration consisted of an Intel Core i7-14700HX CPU, an NVIDIA GeForce RTX 4070 Laptop GPU with 8 GB of dedicated memory, and 32 GB of RAM. The software environment included Python 3.10.15, PyTorch 2.5.1, and CUDA 12.4. The Adam optimizer was employed with default hyperparameters and no weight decay. The initial learning rate was set to 0.0001. A linear learning rate decay strategy was applied starting from the 10th epoch, progressively reducing the learning rate to 0.000001 by the final epoch. Training was performed with a batch size of 4 for 200 epochs. The adversarial training followed a one-to-one update scheme, where the discriminator was updated once for each generator update. No gradient clipping was applied during training. Random seeds were fixed across Python, NumPy, and PyTorch to ensure reproducibility. All timing experiments were conducted on the NVIDIA GeForce RTX 4070 Laptop GPU.

The YOLOv11n detector was trained with default hyperparameters. The training configuration included: input image size of 640, 200 epochs, batch size of 8, optimizer SGD, num workers of 8, and mosaic augmentation disabled after 10 epochs.

### 2.5. Image Quality Assessment

Accurate evaluation of image quality is essential for assessing and optimizing model performance in deblurring tasks. Traditional metrics such as Peak Signal-to-Noise Ratio (PSNR) and Structural Similarity Index Measure (SSIM) are widely used but have clear limitations, particularly in reflecting subjective perception, restoring fine textures, and capturing high-level semantic consistency [23]. Thus, no single metric can fully represent the quality of deblurred images.

To achieve a more comprehensive and objective evaluation in citrus tree motion deblurring, full-reference Image Quality Assessment (IQA) metrics were employed. These metrics jointly measure perceptual fidelity, structural similarity, and statistical characteristics. Prior studies [23,24] analyzed the effectiveness of IQA methods and provided comparative results across visual tasks. Based on these findings, seven representative IQA metrics were selected to form a composite evaluation system, as summarized in Table 1. The score for each indicator is normalized, with values near 1 reflecting higher image quality. The final IQA score is the average of these normalized scores.

**Figure 5:**
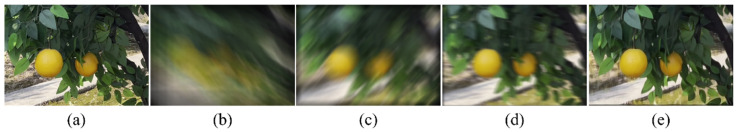
Examples of image quality scores: (**a**) sharp; (**b**) 0.595; (**c**) 0.671; (**d**) 0.765; (**e**) 0.848.

To provide a more intuitive interpretation of the relationship between image quality scores and image clarity, the scores were divided into thresholds as follows: $S\in \left[\left.0\;,\;0.6\right)\right.$ indicates severe blur; $S\in \left[\left.0.6\;,\;0.7\right)\right.$ indicates moderate blur; $S\in \left[\left.0.7\;,\;0.8\right)\right.$ indicates mild blur; and $S\in \left[0.8\;,\;1\right]$ indicates sharp, as shown in Figure 5.

### 2.6. Dynamic Path Scheduling Mechanism

**Figure 6:**
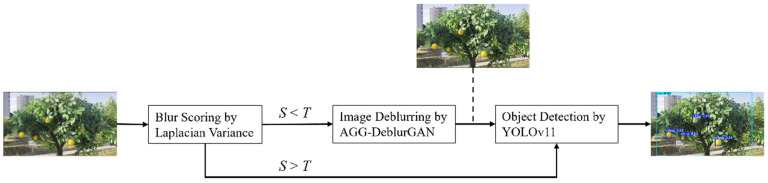
Dynamic path scheduling mechanism based on blur estimation.

To improve processing efficiency, a lightweight blur estimation module was introduced before the deblurring network to enable dynamic path scheduling according to the degree of image blur. Specifically, the system first quantified image sharpness using the variance of the Laplacian method, denoted as *L*. A threshold *T* was then defined to distinguish between sharp and blurred images. If *L* < *T*, the image was considered significantly blurred and was passed through the deblurring network for reconstruction. Otherwise, the image bypassed the deblurring step and was directly forwarded to the object detection model for subsequent processing. It should be emphasized that T is not a universal constant and can be adjusted to meet the requirements of different application scenarios, ensuring adaptability across diverse conditions. The overall system workflow is illustrated in Figure 6.

## 3. Results

### 3.1. Image Deblurring

**Figure 7:**
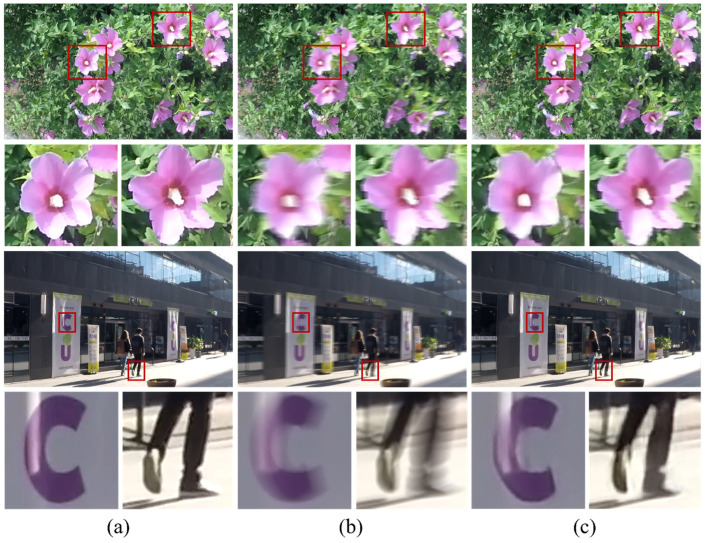
The image deblurring performance of AGG-DeblurGAN on the GoPro dataset: (**a**) sharp; (**b**) blur; (**c**) restoration.

The generalization capability of AGG-DeblurGAN on real-world motion blur was evaluated on the GoPro dataset. As shown in Figure 7, the proposed model effectively recovered fine details, such as object contours and textures, while suppressing blur artifacts in both natural and urban scenes. Quantitative evaluation on the GoPro test set yielded a PSNR of 30.22 dB and an SSIM of 0.928, indicating that AGG-DeblurGAN achieved favorable performance on external real-blur datasets.

On the test set of citrus image dataset, comparative experiments were conducted using the DeblurGAN architecture with different backbone networks. The selected backbones included ResNet, Inception, UNet, GhostNet, MobileNet, and the proposed AGG module. Performance was evaluated from two perspectives: (1) IQA metrics, including DISTS, LPIPS, VSI, FSIM, MS-SSIM, GMSD, and NLPD; and (2) classic image quality indicators such as PSNR and SSIM, along with model complexity metrics including parameter count, FLOPs, memory usage, and runtime speed.

**Figure 8:**
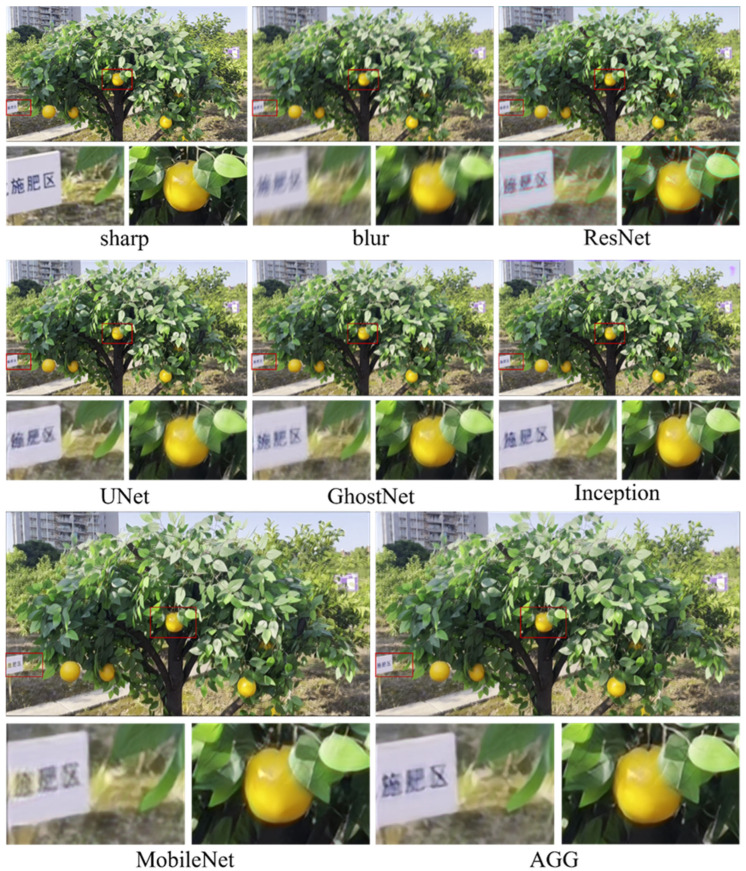
Comparison of deblurring performance for DeblurGAN model with different backbone networks on the self-constructed dataset.

**Figure 9:**
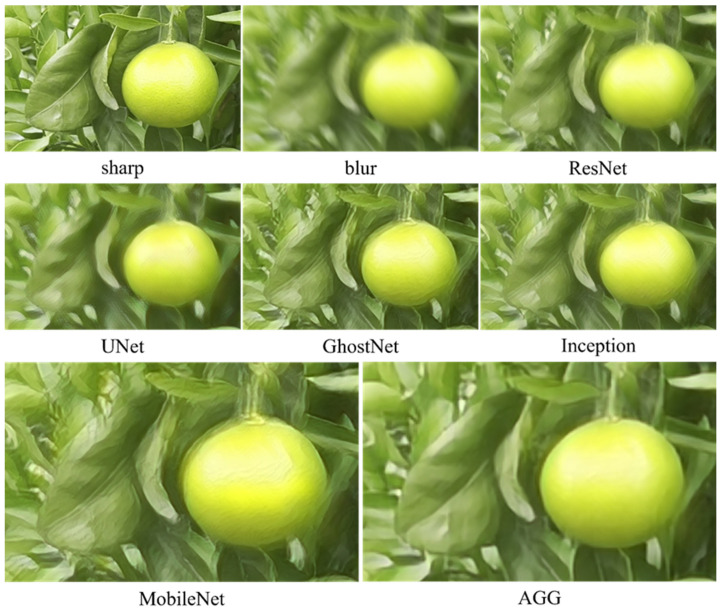
Deblurring results of the real close-up blurred citrus image using different backbone networks, with corresponding sharp images from the same perspective.

Figure 8 shows reconstruction results on synthetic citrus images using different backbones, including the full image and two enlarged regions. Visual inspection revealed clear differences in sharpness, color fidelity, and edge structure. The AGG model preserved higher structural integrity and color consistency across multiple regions: characters in the signage were sharper, fruit textures exhibited well-defined boundaries with smooth light–shadow transitions, and green areas between branches showed no noticeable color bleeding. In comparison, ResNet and UNet produced blurred signage and indistinct fruit boundaries. GhostNet results showed reduced local contrast and slight trailing artifacts in the fruit region, while Inception introduced an unnatural reddish hue in the upper right corner, causing visible color artifacts. MobileNet produced results closest to AGG, with generally high clarity. However, edge leakage was observed in the lower-left corner, where white from the signage blended with the surrounding yellow grass. Overall, AGG delivered more stable performance in both structural recovery and color fidelity. Figure 9 shows the deblurring results on a real close-up blurred citrus image using different backbone networks, with the corresponding sharp image from the same perspective. Compared with the synthetic images, the real image exhibited more complex blur patterns. Visually, ResNet and Inception left residual blur along the fruit boundaries. UNet produced relatively clear fruit regions but failed to preserve hierarchical details in the leaves. GhostNet and MobileNet introduced noticeable color overlapping between fruit and foliage, resulting in a smearing effect. In contrast, the AGG model restored sharper fruit contours and leaf veins while maintaining natural color transitions and smooth shading.

Table 2 presents the comparison of IQA scores for deblurring models with different backbone networks. The AGG model achieved the best performance on most IQA metrics compared to traditional CNN backbones. Specifically, the AGG model obtained a DISTS score of 0.911, outperforming MobileNet by 3.05% and ResNet by 7.42%. For the visually salient sensitive VSI metric, AGG achieved the highest score of 0.971 among all models. In terms of structural similarity, the MS-SSIM, FSIM, and GMSD scores of AGG were 0.863, 0.914, and 0.860, respectively, which were the highest among all backbones. For the statistical modeling metric NLPD, AGG reached 0.697, also the highest score. To comprehensively reflect subjective quality across multiple dimensions, an average of the seven IQA metrics was calculated as an overall score. The AGG model achieved the highest average score of 0.855, representing improvements of 3.64% and 4.78% over traditional networks ResNet and Inception, respectively, and increases of 4.02% and 3.76% compared to lightweight models GhostNet and MobileNet. Statistical analysis was carried out using 95% confidence intervals and paired *t*-tests to compare the AGG model with the other backbones. The AGG model achieved higher IQA scores, with differences that were statistically significant against Test-blur, Inception, and UNet, all with *p* < 0.001. The comparisons with ResNet and GhostNet yielded *p* = 0.015, and the comparison with MobileNet yielded *p* = 0.034. These results indicate that the AGG model achieved competitive performance across the test dataset.

Table 3 presents the comparison of results and parameters for models with different backbone networks. The AGG model achieved a PSNR of 22.35 dB and an SSIM of 0.687, outperforming both the residual-based ResNet and the encoder–decoder UNet in these two metrics. Among lightweight networks, GhostNet and MobileNet achieved PSNR of 21.12 dB and 21.34 dB, and SSIM of 0.609 and 0.626, respectively. AGG further improved the reconstruction accuracy metrics on this basis. Regarding model complexity, AGG had 1.53 million parameters and 1.38 GFLOPs, nearly identical to GhostNet, and significantly lower than traditional convolutional backbones such as Inception and UNet. Additionally, AGG’s memory usage was 75.32 MB, almost the same as GhostNet’s 75.31 MB, and substantially lower than Inception, UNet, and ResNet. These results demonstrate that AGG maintains a lightweight model profile while achieving high reconstruction quality metrics. To evaluate inference efficiency, inference time was measured for 100 runs on a single image (1920 × 1080) using an NVIDIA GeForce RTX 4070 Laptop GPU. The results are reported as mean ± standard deviation. The results showed that AGG-DeblurGAN was the fastest at 58 ms. GhostNet and MobileNet followed with 73 ms and 85 ms, respectively. Inception, UNet, and ResNet required substantially longer inference times of 205 ms, 293 ms, and 384 ms, respectively. For the lightweight AGG network, the deviation was only 1.08 ms, showing stable inference.

**Figure 10:**
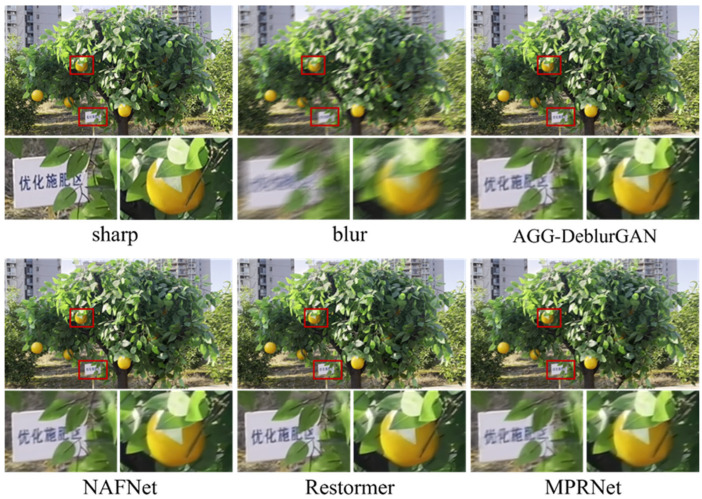
Comparison of deblurring performance for different models on the self-constructed dataset.

**Figure 11:**
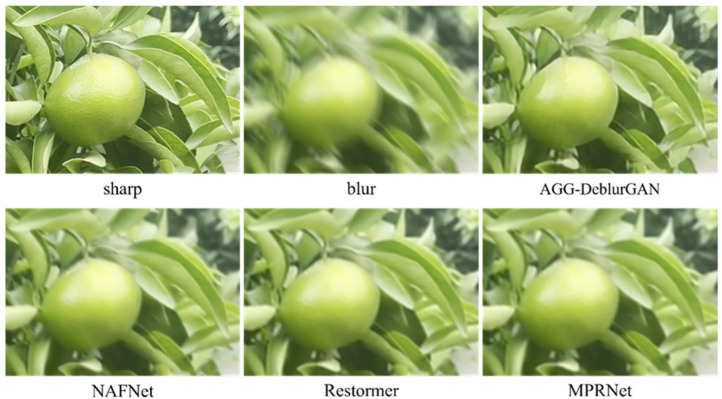
Deblurring results of the real close-up blurred citrus image using different models, with corresponding sharp images from the same perspective.

To further assess the performance of AGG-DeblurGAN, a comparison with representative deblurring models, including DeblurGAN-v2, MPRNet [25], Restormer [16], and NAFNet [26], was conducted on the self-constructed citrus dataset. Table 4 shows quantitative results. Restormer achieved the highest PSNR 23.21 dB and SSIM 0.727, followed by NAFNet. AGG-DeblurGAN obtained PSNR of 22.35 dB and SSIM of 0.687, which were higher than DeblurGAN-v2 and MPRNet. The IQA metric showed the same trend. Restormer achieved 0.872. AGG-DeblurGAN achieved 0.855, exceeding DeblurGAN-v2 at 0.825 and MPRNet at 0.844. Although the restoration performance was not the best, AGG-DeblurGAN exhibited clear advantages in model complexity and inference efficiency. The model contained only 5.24 M parameters and required 58 ms to process a 1920 × 1080 image. In comparison, MPRNet required 1998 ms, Restormer required 1199 ms, and NAFNet required 780 ms under the same conditions. Among all evaluated models, AGG-DeblurGAN achieved the lowest parameter count and the shortest inference time. These results highlight the lightweight design of AGG-DeblurGAN while maintaining competitive restoration performance. Figure 10 shows visual comparisons on synthetic citrus images. All methods achieved effective deblurring. Restormer restored structures closest to the sharp reference, followed by NAFNet. AGG-DeblurGAN and MPRNet showed visually similar results. Figure 11 shows the deblurring results on a real close-up blurred citrus image using different models, with the corresponding sharp image from the same perspective. The real motion blur was more irregular compared with the synthetic images. Restormer provided the sharpest structures and the most natural texture recovery. NAFNet also achieved high clarity, while MPRNet produced reasonable sharpness but left slight residual blur along fruit edges and in some leaf regions. AGG-DeblurGAN restored relatively clear fruit textures and contours, although the sharpness of leaf veins was slightly weaker than that of Restormer. Overall, AGG-DeblurGAN achieved reliable restoration quality on real close-up blurred images.

### 3.2. Ablation Study

The ablation study was performed to evaluate the contributions of the SE module, GHIN module, gating mechanism, and adversarial loss in AGG-DeblurGAN. As shown in Table 5, the complete AGG-DeblurGAN achieved the best results across all metrics, with PSNR of 22.35 dB, SSIM of 0.687, and IQA of 0.855, while maintaining a lightweight design with 5.24 M parameters and an inference time of 58 ± 1.08 ms. Removing any module led to declines in PSNR, SSIM, and IQA. Removing the adversarial loss resulted in the lowest overall performance, with PSNR dropping to 20.81 dB, SSIM to 0.599, and IQA to 0.807, indicating that adversarial loss improved the generator’s performance in GANs. Removing the SE module reduced restoration accuracy, confirming its role in enhancing channel-wise feature representation. The absence of the GHIN module caused a substantial performance drop, highlighting its importance for feature normalization and refinement. Excluding the gating mechanism resulted in moderate degradation, indicating its contribution to adaptive feature modulation. Furthermore, parameter counts remained nearly constant across different configurations, underscoring the lightweight nature of the model.

### 3.3. Citrus Tree Object Detection

To evaluate the impact of image deblurring on downstream task performance, YOLOv11n was employed for citrus tree object detection in orchard scenes. The detector was trained on the sharp images of the citrus dataset, which contained two object categories, namely citrus and tree. For testing, 100 additional citrus tree images that were not included in the training process were used for prediction. Three types of images were employed as input during evaluation: original sharp images, motion-blurred images, and images restored by AGG-DeblurGAN. Detection results under different image qualities are summarized in Table 6. For the mAP metric, sharp citrus images achieved a mAP@0.5 of 0.925 and a mAP@0.5:0.95 of 0.630, while sharp tree images reached 0.995 and 0.868, respectively, serving as upper-bound references. Blurred citrus images dropped markedly to 0.673 at mAP@0.5 and 0.324 at mAP@0.5:0.95, and blurred tree images decreased to 0.967 and 0.741. After restoration by AGG-DeblurGAN, citrus images improved to 0.898 and 0.604, and tree images recovered to 0.995 and 0.842, approaching the sharp image levels. In terms of detection accuracy, blurred citrus images exhibited precision of 0.855, recall of 0.454, and an F1 score of 0.593, reflecting substantial degradation caused by motion blur. After restoration, these values increased to 0.861, 0.803, and 0.831, respectively. For tree detection, blurred images showed 0.955 in precision and 0.900 in recall, resulting in an F1 score of 0.927. The restored group achieved 0.975 in precision and 1.000 in recall, with an F1 score of 0.971, which was close to the sharp baseline of 0.987. The False Negative Rate (FNR) followed the same trend. For citrus, the FNR rose to 0.546 under blur and decreased to 0.197 after restoration, compared with 0.158 for sharp images. For trees, the FNR increased from 0.013 in sharp images to 0.100 under blur, and then decreased to 0.029 after restoration. The mean confidence (mConf) also improved: citrus increased from 0.675 under blur to 0.767 after restoration, approaching 0.787 for sharp images, while tree mConf improved from 0.847 to 0.922, close to the sharp value of 0.928. These results confirm that motion blur significantly reduced detection accuracy, particularly for citrus targets, while restoration by AGG-DeblurGAN effectively recovered detection performance and narrowed the gap with sharp images.

**Figure 12:**
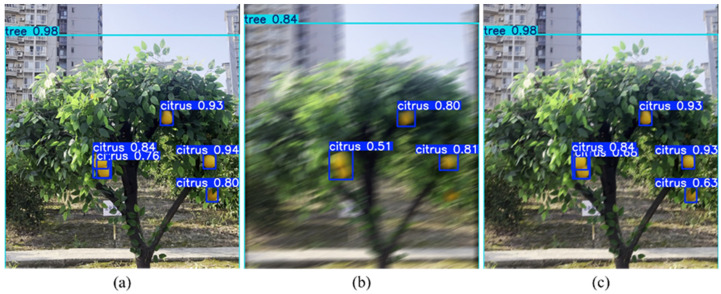
Comparison of object detection results under different image qualities: (**a**) sharp; (**b**) blur; (**c**) restoration.

Figure 12 presents a representative example illustrating the differences in citrus and tree object detection performance across three image types. In the sharp image, object boundaries were clearly defined, allowing YOLOv11n to accurately detect multiple citrus targets with confidence scores ranging from 0.76 to 0.94. The tree category reached a confidence score of 0.98. In contrast, the motion-blurred image exhibited blurred edges and loss of fine details, which resulted in a reduced number of detections and lower confidence scores, dropping to as low as 0.51. Some detection boxes were also misaligned due to degraded visual information. After processing with AGG-DeblurGAN, object detectability was notably restored in several regions. The number of detection boxes increased compared to the blurred image, with many targets being re-identified and confidence scores significantly improved. The confidence for the citrus category increased markedly, closely approximating that of the sharp image, while the tree category regained its confidence score of 0.98. Although some regions still exhibited slightly lower confidence scores compared to the original image, such as 0.63 in the lower-right corner, the overall detection performance was significantly improved.

## 4. Discussion

In fruit tree perception tasks, image blur often leads to unclear object boundaries and loss of texture details, which significantly impairs the accuracy of fruit recognition and detection. Citrus trees are characterized by dense foliage and severe fruit occlusion, while field image acquisition is frequently affected by motion blur caused by camera shake or low illumination. Research on deblurring methods specifically for citrus images remains limited. Shah and Kumar [17] compared several deblurring models on grape images and found that DeblurGAN-v2 achieved the best performance in handling common motion blur. Considering the complexity of agricultural environments and the need for real-time processing, this study introduced lightweight improvements based on the DeblurGAN-v2 architecture. Liu et al. [27] proposed Ghost-DeblurGAN, which employed GhostNet as a lightweight backbone to reduce model parameters and inference time while maintaining competitive detection performance. Inspired by this, the proposed method adopted an eight-layer GhostNet as the encoder backbone, aiming to reduce model complexity while preserving feature extraction capability. In addition, a SE module was integrated to enhance the response of informative channels and improve the feature representation. For normalization, Chen et al. [28] introduced Half-Instance Normalization (HIN), which demonstrated superior performance in image restoration tasks. Wang et al. [22] proposed a gating mechanism that modulates the normalization results through learnable gating parameters. Based on this, a gated variant, the Gated Half-Instance Normalization (GHIN) module, was incorporated to dynamically modulate normalization intensity, thereby adapting to complex textures and spatial variations in citrus images. In summary, this study introduced AGG-DeblurGAN, a lightweight generative adversarial network that incorporates GhostNet, SE modules, and GHIN modules for citrus image deblurring. While the method improved downstream object detection performance, its evaluation was limited to a specific dataset and application scenario. Broader validation across diverse environments will be necessary to confirm its generalizability.

To the best of our knowledge, no publicly available real motion-blurred dataset provides strictly aligned pairs of sharp and blurred images. Unlike datasets for deraining, dehazing, or denoising, motion blur datasets face inherent challenges in capturing perfectly matched pairs due to device limitations and the physical principles of motion. Synthesizing blurred images from sharp ones has therefore become a practical solution. The widely used GoPro dataset generated blurred images by averaging 15 consecutive sharp frames. Yun et al. [19] built motion blur datasets for weeds using kernel trajectory to generate random and nonlinear motion blur. In this study, blurred citrus images were synthesized from sharp images using randomly motion trajectories combined with convolution. A qualitative comparison with the GoPro dataset confirmed the realism and usability of the proposed method. Of course, studying motion blur datasets with matched real sharp and blurred images is also important.

Image quality assessment plays a crucial role in image restoration tasks, particularly for quantifying differences between restored images and their reference counterparts. PSNR and SSIM are currently the most widely used evaluation metrics. PSNR measures numerical fidelity based on pixel-wise errors and is computationally simple. SSIM evaluates image quality by comparing luminance, contrast, and structural information, offering a more perceptually relevant assessment. However, both metrics rely heavily on precise alignment with reference images and are highly sensitive to differences in complex or visually similar textured regions, for example, different cropped areas of a pebble cluster [24]. In this study, the highest PSNR and SSIM values achieved by AGG-DeblurGAN for citrus tree deblurring were 22.35 dB and 0.687, which fall below the commonly accepted thresholds for clear images (PSNR > 28 dB and SSIM > 0.85). This gap suggests that, although the restored images often appeared visually similar to the ground truth, the model may not capture all structural and perceptual details required by standard quantitative metrics. The inconsistency between subjective perception and objective measures highlights the need for more comprehensive evaluation criteria and further refinement of the model. Prior studies have reported similar challenges, as complex natural textures and overlapping multi-scale objects reduce the reliability of traditional metrics. For example, DeblurGAN-v2 achieves PSNR and SSIM values of 29.55 and 0.934 on the synthetic GoPro dataset but declines to 20.63 and 0.7185 on the PlantVillage dataset with diverse plant structures [18]. On a private orchard dataset, these metrics further decrease to 21.95 and 0.731 [29], underscoring the strong influence of environmental complexity on evaluation outcomes. To address these inconsistencies, this study incorporated multiple full-reference FR-IQA metrics, including perception-based (DISTS, LPIPS, VSI), structure similarity-based (MS-SSIM, FSIM, GMSD), and statistical modeling-based (NLPD) measures. While these complementary perspectives provide a more nuanced assessment of image restoration quality, they also highlight that no single metric can fully reconcile subjective perception with objective quantification in complex natural scenes.

Although AGG-DeblurGAN achieved lower restoration accuracy than recent SOTA models such as Restormer, NAFNet, it offered a clear efficiency advantage. Runtime comparisons should nevertheless be interpreted with caution, as they depend heavily on hardware and input resolution: NAFNet reported 230 ms on 1280 × 720 images with a 2080Ti GPU, and Restormer on 256 × 256 inputs. These differences indicate that model speed is influenced by implementation details. The evaluation results showed that AGG-DeblurGAN had lower complexity and higher computational efficiency. This property makes it suitable for real-time or constrained agricultural applications.

To further examine the impact of image deblurring on downstream task performance, the proposed method was evaluated on a target detection task in a citrus orchard scenario, with a detailed analysis of detection metric variations across different image quality conditions. Building on these observations, a dynamic path scheduling mechanism driven by blur-level assessment was developed, as illustrated in Figure 6. A lightweight blur detection module was introduced at the front end to determine whether deblurring should be applied based on image clarity. This approach prevented unnecessary processing of sharp images and reduced inference latency by conserving computational resources. While this design provides a task-aware strategy that links image restoration to system efficiency, several trade-offs should be noted. The reliance on Laplacian variance for blur quantification, although computationally efficient, may not capture all forms of degradation, and the choice of detection threshold requires careful adjustment for different application settings. These considerations suggest that, although the mechanism is promising for resource-constrained environments such as agricultural robots, further refinement and validation across diverse conditions are needed to confirm its broader applicability.

Beyond the reported results, several aspects remain unexplored and should be acknowledged as limitations of this work. First, the study focused exclusively on motion blur caused by camera or object movement, whereas other types of degradation, such as defocus blur or depth-of-field artifacts, were not investigated. These forms of blur may pose distinct challenges and could affect the generalizability of the proposed approach. Second, although the GHIN module was designed to enhance feature normalization and improve restoration quality, its behavior in highly textured or cluttered regions has not been fully examined, raising the possibility of unintended artifacts. Third, the evaluation was conducted primarily on a citrus orchard dataset; thus, the performance of AGG-DeblurGAN in different agricultural settings or under varying environmental conditions remains uncertain. Fourth, while the integration of multiple perceptual metrics provided a more nuanced assessment of deblurring quality, these metrics may still not fully capture human visual perception. This limitation suggests the need for further investigation using subjective evaluation protocols. Finally, practical deployment challenges should also be considered, such as robustness under diverse lighting and weather conditions. Addressing these issues in future studies will be essential for achieving a more comprehensive validation of the method and ensuring its applicability in diverse real-world scenarios.

Future work may proceed in several directions. First, blur estimation strategies that combine multiple cues, such as frequency-domain features, texture descriptors, and deep blur perception networks, could be explored to improve the accuracy and robustness of blur assessment. Second, the development of an end-to-end blur assessment module that jointly optimizes blur recognition and target detection represents a promising direction, although challenges related to model complexity and training stability remain. Addressing these aspects would enhance system performance and improve the generalization of deblurring-assisted vision systems in diverse agricultural scenarios.

## 5. Conclusions

This study addressed the problem of non-uniform motion blur caused by motion and occlusion in citrus tree images by proposing a lightweight end-to-end image deblurring network, AGG-DeblurGAN, which was applied as a preprocessing step for target detection tasks. The model integrated a GhostNet backbone, SE attention mechanism, and GHIN module to balance feature representation capability with computational efficiency. A full-reference IQA methodology was introduced, which balanced subjective perceptual features and structural similarity differences, providing a more comprehensive and accurate evaluation of deblurring performance. Experimental results demonstrated that AGG-DeblurGAN achieved superior performance across multiple image quality metrics and significantly improved YOLOv11n object detection accuracy, effectively restoring image details, enhancing object visibility, and increasing detection confidence in complex natural environments. Additionally, a Laplacian variance-based blur detection module was incorporated to rapidly distinguish between sharp and blurred images, enabling dynamic scheduling of deblurring execution and further improving system efficiency and practicality. Overall, the proposed lightweight deblurring method was able to restore image quality and improve detection precision. It can serve as a reference for improving image preprocessing and detection performance in agricultural vision systems. Future work will focus on non-uniform blur recognition, multi-object classification, and end-to-end joint optimization to adapt to increasingly complex field environments and real-time processing requirements.

## Figures and Tables

**Table 1:** Image Quality Assessment metrics.

Name	Method	Type
DISTS	Deep Image Structure and Texture Similarity	Perceptual features
LPIPS	Learned Perceptual Image Patch Similarity	Perceptual features
VSI	Visual Saliency-Induced Index	Perceptual features
MS-SSIM	Multi-Scale Structural Similarity	structural similarity
FSIM	Feature Similarity Index	structural similarity
GMSD	Gradient Magnitude Similarity Deviation	structural similarity
NLPD	Normalized Laplacian Pyramid Distance	statistical modeling

**Table 2:** Comparison of IQA Scores for Deblurring Models with Different Backbone Networks. (**a**). Comparison of IQA scores for DeblurGAN model with different backbone networks on the self-constructed dataset (95% CI). (**b**). Comparison of IQA scores for DeblurGAN model with different backbone networks on the self-constructed dataset (95% CI).

(**a**)				
**Networks**	**DISTS**	**LPIPS**	**VSI**	**MS-SSIM**
Test-blur	0.723 ± 0.012	0.638 ± 0.011	0.868 ± 0.010	0.472 ± 0.032
ResNet	0.848 ± 0.005	0.740 ± 0.004	0.961 ± 0.003	0.817 ± 0.010
Inception	0.854 ± 0.006	0.729 ± 0.005	0.956 ± 0.003	0.802 ± 0.011
UNet	0.853 ± 0.005	0.740 ± 0.005	0.959 ± 0.003	0.812 ± 0.014
GhostNet	0.881 ± 0.004	0.724 ± 0.004	0.960 ± 0.003	0.810 ± 0.008
MobileNet	0.884 ± 0.004	0.729 ± 0.004	0.961 ± 0.003	0.809 ± 0.009
AGG	0.911 ± 0.003	0.766 ± 0.004	0.971 ± 0.002	0.863 ± 0.008
(**b**)				
**Networks**	**FSIM**	**GMSD**	**NLPD**	**Score**
Test-blur	0.679 ± 0.018	0.783 ± 0.008	0.546 ± 0.010	0.673 ± 0.012
ResNet	0.891 ± 0.006	0.847 ± 0.002	0.673 ± 0.006	0.825 ± 0.003
Inception	0.881 ± 0.006	0.836 ± 0.003	0.654 ± 0.007	0.816 ± 0.006
UNet	0.885 ± 0.007	0.843 ± 0.004	0.665 ± 0.008	0.822 ± 0.006
GhostNet	0.889 ± 0.005	0.837 ± 0.002	0.655 ± 0.005	0.822 ± 0.004
MobileNet	0.891 ± 0.005	0.838 ± 0.002	0.654 ± 0.006	0.824 ± 0.003
AGG	0.914 ± 0.004	0.860 ± 0.003	0.697 ± 0.007	0.855 ± 0.004

**Table 3:** Comparison of results for the DeblurGAN model with different backbone networks on the self-constructed dataset.

Networks	PSNR	SSIM	IQA	Params (M)	Flops (G)	Memory (MB)	Inference Time (ms)
Test-blur	18.07	0.404	0.673	**/**	**/**	**/**	**/**
ResNet	21.10	0.602	0.825	7.84	32.71	346.09	384 ± 1.95
Inception	20.48	0.572	0.816	35.84	17.57	214.38	205 ± 2.81
UNet	21.07	0.603	0.822	51.29	21.40	280.99	293 ± 1.98
GhostNet	21.12	0.609	0.822	1.52	1.38	75.31	73 ± 1.28
MobileNet	21.34	0.626	0.824	2.11	2.74	119.92	85 ± 1.19
AGG	22.35	0.687	0.855	1.53	1.38	75.32	58 ± 1.08

**Table 4:** Comparison of the results for different models on the self-constructed dataset.

Method	PSNR	SSIM	IQA (95% CI)	Params (M)	Inference Time (ms)
DeblurGAN-v2	21.10	0.601	0.825 ± 0.003	28.84	384 ± 1.95
MPRNet [25]	22.29	0.673	0.844 ± 0.012	20.1	1998 ± 3.71
Restormer [16]	23.21	0.727	0.872 ± 0.008	16.13	1199 ± 3.79
NAFNet [26]	22.64	0.703	0.860 ± 0.010	67.89	780 ± 3.27
AGG-DeblurGAN	22.35	0.687	0.855 ± 0.004	5.24	58 ± 1.08

**Table 5:** Comparison of the results for different modules on the self-constructed dataset.

Model	PSNR	SSIM	IQA (95% CI)	Params (M)	Inference Time (ms)
AGG-DeblurGAN	22.35	0.687	0.855 ± 0.004	5.24	58 ± 1.08
Remove adversarial loss	20.81	0.599	0.807 ± 0.005	5.24	58 ± 1.07
Remove SE module	22.06	0.674	0.849 ± 0.005	5.23	54 ± 1.67
Remove GHIN module	21.15	0.611	0.819 ± 0.006	5.24	53 ± 1.10
Remove Gating mechanism	21.45	0.633	0.823 ± 0.004	5.24	53 ± 1.12

**Table 6:** Detection performance of sharp, blurred and restored images by AGG-DeblurGAN.

Source	Class	mAP@0.5	mAP@0.5:0.95	Precision	Recall	F1	FNR	mConf
Sharp	citrus	0.925	0.630	0.868	0.842	0.855	0.158	0.787
	tree	0.995	0.868	0.982	1.000	0.987	0.000	0.928
Blur	citrus	0.673	0.324	0.855	0.454	0.593	0.546	0.675
	tree	0.967	0.741	0.955	0.900	0.927	0.100	0.847
Restore	citrus	0.898	0.604	0.861	0.803	0.831	0.197	0.767
	tree	0.995	0.842	0.975	1.000	0.981	0.000	0.922

## Data Availability

The original contributions presented in the study are included in the article, further inquiries can be directed to the corresponding author.
